# Rural citizen-patient priorities for healthcare in British Columbia, Canada: findings from a mixed methods study

**DOI:** 10.1186/s12913-021-06933-z

**Published:** 2021-09-18

**Authors:** Jude Kornelsen, Christine Carthew, Kayla Míguez, Matilda Taylor, Catherine Bodroghy, Kathryn Petrunia, Delia Roberts

**Affiliations:** 1 Centre for Rural Health Research, Department of Family Practice, University of British, 3rd Floor David Strangway Building, University of British Columbia, 5950 University Boulevard, British Columbia V6T 1Z3 Vancouver, Canada; 2 Rural Patient Partner, British Columbia, Canada

**Keywords:** Rural Health, Patient-Oriented Research, Community Participation, British Columbia

## Abstract

**Background:**

The challenge of including citizen-patient voices in healthcare planning is exacerbated in rural communities by regional variation in priorities and a historical lack of attention to rural healthcare needs. This paper aims to address this deficit by presenting findings from a mixed methods study to understand rural patient and community priorities for healthcare.

**Methods:**

We conducted a provincial survey of rural citizens-patients across British Columbia, Canada to understand their most pressing healthcare needs, supplemented by semi-structured interviews. Survey and interview participants were asked to articulate, in their own words, their communities’ most pressing healthcare needs, to explain the importance of these priorities to their communities, and to offer possible solutions to address these challenges. Open-text survey responses and interview data were analyzed thematically to elicit priorities of the data and their significance to answer the research questions.

**Results:**

We received 1,287 survey responses from rural citizens-patients across BC, 1,158 of which were considered complete. We conducted nine telephone interviews with rural citizens-patients. Participants stressed the importance of local access to care, including emergency services, maternity care, seniors care, specialist services and mental health and substance use care. A lack of access to primary care services was the most pronounced gap. Inadequate local health services presented geographic, financial and social barriers to accessing care, led to feelings of vulnerability among rural patients, resulted in treatment avoidance, and deterred community growth.

**Conclusions:**

Two essential prongs of an integration framework for the inclusion of citizen-patient voices in healthcare planning include merging patient priorities with population needs and system-embedded accountability for the inclusion of patient and community priorities.

**Supplementary Information:**

The online version contains supplementary material available at 10.1186/s12913-021-06933-z.

## Background

In recent decades, there has been recognition of the importance of a broad coalition of key stakeholders in healthcare decision-making and the attendant move from an administratively-oriented process to one that prioritizes diversity of voices. Decision-making tables now include participation from those providing and receiving care alongside others with a vested interest in health service delivery such as industry. Broad-spread recognition of the importance of healthcare users’ input has gained so much traction that many jurisdictions have instituted mechanisms to facilitate such involvement from an individual committee level (e.g., British Columbia Patients as Partners^1^) [1] to a systemic level through prioritized patient-oriented research [2]. In British Columbia (BC), Canada, citizen-patient participation in healthcare decision-making, planning and research takes many forms and occurs at varying levels (locally, regionally, provincially). For instance, the Patient Voices Network operates at a provincial level to pair patient partners with healthcare stakeholders including researchers seeking to incorporate patient perspectives into their work [3]. Likewise, BC’s Regional Health Authorities have responsibilities to engage citizens-patients to plan and deliver health services that satisfy population needs in their respective regions [4]. Engagement opportunities are unique to each Health Authority and might involve focus groups, surveys and workshops, presentations to municipal councils and community organizations, and participation on advisory committees.

What is less clear, however, is the agency of citizens-patients to be proactively involved in shaping strategic agendas as opposed to responding to health system priorities. In BC, there are few, if any, established mechanisms to proactively gather citizen-patient input for priority setting activities. A further challenge of proactivity is finding these opportunities for involvement in a healthcare system that is distributed and siloed. This is compounded by the diversity of citizen-patient voices and the danger in assuming homogeneity within this group.

Including rural citizen-patient voices specifically in healthcare planning is an added challenge due to regional variation in priorities and the historical lack of attention to rural healthcare needs. Further, the capacity of rural citizens-patients to organize beyond a community level is hampered by the tyranny of geography and the very definition of rural (low population density and isolation), often leading to participation from too few citizens. This is not to suggest a lack of local community advocacy for health services, but instead a lack of proactive involvement.

British Columbia is Canada’s third largest province with a land mass of nearly 950,000 square kilometres. Despite its expansive geography, the majority of the province’s population is concentrated in urban areas that account for 5 % of the land base [4]. Meanwhile, 13.6 % of the population is located in non-urban settings that encompass 95 % of the land area [5]. It is unsurprising then that rural BC communities are often small and dispersed [4].

British Columbia’s rural residents are older than their urban counterparts and as populations age, their need for services including healthcare increases [6]. Additionally, rural residents experience poorer socioeconomic status, including lower educational attainment, higher incidences of unemployment and lower average earnings, and poorer health status, including higher incidences of some chronic diseases, poorer perinatal health outcomes, and higher rates of all-cause mortality compared with urban dwellers [4, 6]. Nonetheless, low population density in rural areas in combination with the vast geographical landscape, make it difficult to sustain specialist services and hamper access to primary care [6, 7]. Inclement weather, mountainous terrain, reliance on ferry schedules and a lack of land-based intra- and inter-community public transportation options in many rural areas compound these barriers to accessing care.

The organizing principle of ‘community’ in the rural context is important as isolated geographies often intensify collective culture and lead to a strong sense of identity and unity through locality. This sense of belonging in many communities, alongside the shared priorities that are location-specific (e.g., lack of local access to emergency care), give rise to the importance of recognizing *community* along the citizen-patient continuum. Because of this, the description of our engagement framework is citizen-patient-community (CPC).

The Canadian Strategy for Patient-Oriented Research (SPOR) represents a coalition of federal, provincial and territorial partners with the mandate to integrate patient voices into research and subsequently, to ensure that patient priorities are reflected in policy and practice. The Rural Evidence Review project has funding under SPOR to capture and catalogue rural BC citizen-patient priorities for health services, to translate these priorities into research questions for evidence synthesis on best practices for health service delivery, and to move the findings forward into provincial and regional policy discussions. We do this through a ‘rural lens’ that seeks to understand and acknowledge the nuances of rurality including the implications of low population density and isolation, and the differential impacts that policies (and evidence) may have on health planning. The lodestar for this work is citizen-patient-community articulated priorities gathered and presented in a way that appreciates inter-community differences but also seeks commonalities that can be understood at a provincial level. This paper presents such thematic priorities.

Although there are challenges in rural CPC engagement and mobilization, it remains a much simpler task than actioning the articulated priorities into health policy change. Without overcoming this enduring stumbling block, however, no meaningful CPC-responsive system improvements can occur and we will fall short of our commitment to CPC inclusion in health planning. Articulating CPC priorities is a tentative first step toward actualizing this agenda.

There has been a recent proliferation of patient priority setting studies for both health research and health system planning purposes. A recent rapid review of the patient engagement and priority setting literature retrieved 70 articles describing public and patient priority setting through diverse engagement processes and activities, including surveys [8]. However, a majority of studies have focused on populations defined by specific health conditions [8, 9] and priorities for particular care types [10, 11], and it is common for the activities to bring forward a pre-determined (often, by expert opinion) set of potential topics to patients for them to prioritize rather than for patients and the public to self-identify their own priorities for care [8]. Moreover, there was a dearth of literature exploring healthcare priority setting through a rural lens.

 We did identify a small number of publications that explored healthcare priorities broadly with a rural focus, which allowed respondents to articulate their own priorities for care [12–14]. Rich et al. [12] reported data from the Australian Rural Mental Health Survey and in particular, responses to the open-ended question ‘What health services would you like to see the local district providing that are currently not available in your area?’. However, the primary objective of this research was not to articulate priorities for care but instead to understand the utility of automated programs to supplement the analysis of open-ended survey responses and to identify the characteristics of participants who respond to open-text survey questions [12]. Nonetheless, the authors reported that, while less in-depth than interview data, open-ended survey questions provided enough information to ascertain a broad overview of the rural health service priorities identified by the sample [12]. Likewise, Panelli et al. [14] investigated the intersection of policy and politics with lived experiences of healthcare in New Zealand (NZ) by comparing the 1999 NZ Rural Health Policy with rural citizens’ experiences of accessing healthcare as articulated through a community-led, nation-wide survey. Similarly to our study, this study offers detailed insight into the local healthcare priorities and challenges of a sizeable sample of rural residents (1,240 total responses with 48 % of respondents answering open-text questions) [14].

There are a multitude of methodological approaches that have been used to engage patients in healthcare priority setting. However, there doesn’t appear to be a single, best method and instead, the choice of method might be best guided by the nature and objective of the research itself [15]. In the present case, the online survey method was chosen for its utility to elicit an overview of priorities from a large subset of the population [12, 16] – residents across rural and remote BC communities – in a pragmatic and cost-effective way. Follow-up semi-structured telephone interviews were offered to supplement the online survey, offering opportunity for in-depth reflection and discussion regarding rural citizens’ healthcare priorities and needs [16].

## Methods

### Survey

In January 2019, the Rural Evidence Review project launched an online survey (see Additional file 1) to learn from rural citizens-patients across BC about their most pressing healthcare needs. Respondents were asked to articulate, in their own words, their communities’ most pressing healthcare needs, to explain the importance of these priorities to their communities, and to offer possible solutions to address these challenges. Additionally, several demographic questions were included in the survey to solicit each respondent’s age, sex, place of residence, length of time living in their community, and whether they were a paid healthcare provider. Completion of the online survey indicated consent to participate, which was explained to respondents on the survey’s information landing page. The online survey was hosted on the University of British Columbia Survey Tool, provided by Qualtrics.

The survey was shared to Rural Practice Subsidiary Agreement (RSA)^2^ communities through local newspapers and radio stations, community Facebook groups, local elected council and local Chambers of Commerce, where contact information was publicly available. Information on the survey, including the research objective, how to participate and contact information for the research team, were sent to key contacts (e.g., newspaper editors and reporters, mayor and council, and Facebook group administrators) by email or Facebook Messenger, with the request to share this information to their constituents. All residents of BC RSA communities were eligible for participation in the online survey.

The survey data were analyzed using both qualitative and quantitative methods. Prism 8 statistical software was used to generate descriptive statistics for the responses to demographic questions from non-healthcare providers. Open-text responses from non-healthcare providers were analyzed thematically using NVivo 11 qualitative data analysis software.

The complete survey responses up to July 10 2019 from non-healthcare providers were included for analysis (statistical and thematic). A response was considered complete and eligible for analysis if one or more of the open text questions, ‘What are the most important healthcare priorities in your community?’ (Q7), ‘Why are these priorities important in your community?’ (Q8), and ‘What do you think could be done to improve healthcare in your community?’ (Q9), were answered.

Data from open-ended questions were subjected to thematic analysis. One member of the research team (KM) read through and familiarized themselves with all open-text survey responses. The data were then uploaded to NVivo 11 software wherein open-text responses were inductively coded using thematic analysis methodology [17]. A preliminary codebook was created and subsequently reviewed by three members of the research team (KM, JK, CC). Feedback was incorporated. A final codebook was then established, reviewed and agreed upon by the research team (KM, JK, CC). The final codebook guided the coding of the complete data set for each open-ended question. The coded data were then analyzed by one member of the research team (JK) to establish main and sub-themes of the data and their significance to answer the research questions. The responses to open-text questions were analyzed separately, with the exception of questions 7 and 8 as the responses to these two questions were often linked and benefited from being read and analyzed concurrently. All open-text responses were paired to the respondents’ communities, to allow for the subsequent analysis of responses on a community-basis.

### Interviews

Semi-structured telephone interviews were offered in addition to the survey. Information on the interviews was shared at the same time and through the same means as the survey. Individuals interested in participating in a telephone interview, as an alternative or in supplement to the survey, were asked to contact the research team by email or telephone. One member of the research team (CC) then followed up to schedule and conduct the interviews. Participants provided written consent prior to the start of the interviews, oral consent at the start of the interviews, or both. The open-text questions included in the survey instrument, including ‘What are the most important healthcare priorities in your community?’ (Q7), ‘Why are these priorities important in your community?’ (Q8), and ‘What do you think could be done to improve healthcare in your community?’ (Q9), served as the basis for the interviews. Although, the semi-structured and in-depth nature of the interviews allowed for flexibility to modify, expand and iterate on the interview questions to best uncover the nuances of participants’ priorities for care. Interviews were audio recorded and supplementary notes were taken by the interviewer. Audio recordings were transcribed verbatim by Scriptastic Transcription Services.

Likewise, interview data from non-healthcare providers were thematically analyzed. One member of the research team (MT) listened to the audio recordings of the interviews and read through the interview transcripts to familiarize themselves with the data and to gain a sense of the entirety of the data. The interview transcripts were then uploaded to NVivo 11 software, where the data were deductively and inductively coded. The final coding scheme derived from the analysis of open-text survey responses served as provisional parent nodes, and emergent codes were added as necessary to accommodate for ideas and patterns unique to the interview data. The codes were then refined by reviewing all of the coded data to determine fit to previously established code definitions (i.e., to look for similarities and differences in the two data sets) and to check for redundancies. A preliminary codebook was reviewed by three members of the research team (MT, JK, CC) and feedback was incorporated. A final codebook was established, agreed upon by three members of the research team (MT, JK, CC) and applied to the interview data. The coded data were then analyzed by one member of the research team (JK) to establish main and sub-themes of the data, their congruency with the survey data findings, and their significance to answer the research questions.

 All research methods were carried out in accordance with relevant guidelines and regulations.

It is important to note that the research study was originally conceptualized as a quality improvement initiative (i.e., not for research purposes) with the objective to understand rural community priorities for care, that would then inform the direction of the project in terms of what we studied. A short time after the survey was launched, however, we recognized the importance of the information that we collected, as evidenced by the high response rate and richness of the data. At this time, we paused data collection and applied to the University of British Columbia’s (UBC) Behavioural Research Ethics Board (BREB) to use the data for research purposes, including the data we received prior to ethics approval and all data received after ethics approval. Our application to UBC’s BREB was approved (H19-00254).

Our due diligence involved sharing an update message to all sources that were previously contacted to recruit participants (e.g., local newspapers and radio stations, Chambers of Commerce, elected council, etc.), informing them of our interest in using the data for research purposes and emphasizing that in no way would the data be identifiable to any participant. These sources were asked to share the update message with their constituents.

## Results

In total, we received 1,463 survey responses; 1,287 responses from rural citizens-patients (non-healthcare providers), 132 responses from healthcare providers, and 44 responses from individuals who chose not to specify whether they were a healthcare provider. 1,158 of the responses from non-healthcare providers were considered complete. Additionally, 14 individuals contacted the research team with interest in participating in an interview either instead of or in follow-up to their participation in the online survey. All were eligible to participate and invited to interview. We conducted nine interviews with non-healthcare providers and one interview with a healthcare provider. We were unable to reach the remaining four individuals after their initial expressions of interest. We did not ask interview participants whether they completed or intended to complete the online survey.

Here we report the demographic profile of non-healthcare provider survey respondents, as well as findings from the open-text survey and interview data from non-healthcare providers. The findings of the interview data were congruent with the findings of the open-text survey data and therefore, we integrated the findings of both data sets and present them together, below. The survey and interview data from healthcare provider respondents will be analyzed and reported on separately.

### Demographics of survey respondents

The survey respondents represented 186 discrete communities across rural British Columbia. All five of BC’s Regional Health Authorities are represented in the data, with the majority of participants representing communities within the Northern Health and Interior Health Authorities (Table 1: Respondents by Health Authority). The average age of respondents was 56.3 years, with a range of 18 to 91 years (Table 2: Respondents by Age Range). The average length of time that respondents lived in their communities was 21.7 years, with a range of 1 month to 83 years. We heard from 911 female and 233 male respondents, while one participant reported their sex as ‘other’, eight participants selected ‘prefer not to say’, and five respondents did not answer this question.

**Table 1:** Respondents by Health Authority

Health Authority	Total Respondents (No.)	Female Respondents (No.)	Male Respondents (No.)	Other Respondents^a^ (No.)	Mean Age (Years)
*Interior Health Authority*	642	500	135	7	58.9
*Northern Health Authority*	369	293	73	3	50.6
*Vancouver Island Health Authority*	66	55	10	1	59.3
*Vancouver Coastal Health Authority*	62	47	13	2	59.2
*Fraser Health Authority*	13	12	1	0	56.4
*Not Reported*	6	4	1	1	60.5

Footnote: ^a^Other respondents include those who reported their sex as ‘other’, who preferred not to report their sex, and who did not answer the question regarding their sex

**Table 2:** Respondents by Age Range

Age Range (Years)	Number of Respondents
80+	47
70–79	178
60–69	315
50–59	194
40–49	161
30–39	142
20–29	52
< 20	2
Not Reported	67

### Health care priorities

Participants were asked to identify the most important healthcare priorities for their communities along with a rationale for their responses. The overarching theme from the data was the importance of access to care for rural areas, with consideration for the unique demographic, geographical and social contexts of rural communities and the importance of access based on discrete care needs (e.g., maternity or surgical care).

#### The context of rural care

Almost all participants emphasized the geographic reality of rural BC, marked by distance to the next, larger medical centre ranging from just under one hour to more than four hours of travel. For most respondents, travel time was compounded by poor travel conditions, especially in inclement weather (e.g., winter weather conditions), which led to further isolation for many months of the year. Many participants noted that they were from island communities, dependent on ferries to access some, if not all, services.

The challenge of distance to care was exacerbated for many by local demographic characteristics reflecting population segments including for instance, children and youth, new families, and seniors. As one participant said,

*[Our community] is a family town, with many elderly in need of care, mothers who need as much support as they can get, and troubled youth, people with mental illnesses, and people suffering from addictions to drugs and alcohol.*

Many respondents noted that population demographics, notably size, change dramatically during periods of high tourism (e.g., summertime) leading to increased demand for local health services and reduced access for locals.

There was general consensus on the diversity across rural communities and the concomitant need for diverse health services. Along with isolation and diversity, however, the reality for many rural communities is an expanding population with decreasing local access to health services.

#### The importance of access to care

Many participants described difficulties traveling to access care, including the time required, lack of transportation options and lack of back-up if they themselves are in care-giving roles. The financial impact of traveling to care including transportation, accommodation and lost wages was particularly salient. Beyond the impact on the individual patient, this was seen to affect patients’ families and their extended community supports, who may need to take time away from work and other activities to accompany the patient. In contrast, many participants noted the disadvantage of people who did not have a support network.

There were discrete, experience-based comments regarding the need for improved access to particular care modalities. These are presented in order of frequency with which they were reported in the data, below.

##### Primary care

Local access to primary care was seen by many participants to be both foundational to good healthcare and also lacking in many communities. Several respondents noted a high rate of unattached patients in their communities. This was recognized by many as a product of challenges to recruitment and retention, with an awareness that rural communities compete with larger, urban centres for the same providers. One respondent noted, “we lose, every time.” Many participants recognized that a key consideration for both recruiting and retaining providers was to attract those “who are part of the fabric of the community, ones who would be involved in the soccer team and church groups” and those who “appreciate what a small community has to offer”. This idea was reiterated by several interview participants, who recognized the difficulty of attracting physicians to rural communities. These individuals noted a lack of incentives for physicians to practice “anywhere … but with his buddies in Vancouver”. Likewise, other participants recognized the draw of larger centres.

Many participants noted a lack of continuity of care. As one participant described, “We have doctors, [nurse practitioners] and locums who [each] have a different health plan for you…”. The lack of continuity of care led to a lack of capacity to build relationships, leading to parts of care “falling between the cracks” (e.g., test results).

For those who did have local care providers, shortages were marked by long wait-times and the attendant consequence of “being forc[ed] to use the ER”. This was seen by many to lead directly to poorer health outcomes but also system inefficiencies: “most people in our town are relying on the ER for prescription refills, colds, etc.”. Some respondents suggested that a walk-in clinic would alleviate at least some of these issues.

##### Seniors care

Many survey respondents prioritized seniors care, alongside the observation that many rural communities have an aging population and significant in-migration following retirement and due to a desire for decreased cost of living. Participants recognized the challenges that rurality poses for an elderly population including reduced driving capacity to access healthcare appointments locally and in referral communities, and a lack of access to local home supports and long-term care facilities. Most respondents agreed that ideally rural seniors should be supported to age in place, both for increased quality of life and reduced health system costs. Participants felt that this would have a positive impact on individual patients and also lead to a “more wholesome community.” As one participant noted,

In order to grow, the aging must be around to share their expertise with the families that are courageous enough to stick around and bring new life into the community.

Where seniors care is not available locally, participants pointed out that the impact of separation from family and community could be significant. It was noted too, however, that the alternative to leaving one’s community for care was the inappropriate placement of seniors in non-acute hospital beds, which was not desirable from either an individual or system perspective. Some respondents were critical of “stringent rules around home care” and the consequence of “very unhappy seniors living in institutional care homes”. For those who wanted a supported setting, costs were reported as a substantial barrier.

Several respondents noted that the challenge of access to appropriate local home supports and long-term care facilities was not restricted to seniors, but affected others requiring end-of-life care or severe chronic disease management.

##### Emergency care

Access to emergency care was described as paramount by many respondents. Again, access was compromised in many instances by inclement weather and the geographical reality of rural BC. This was described in the context of local access to emergency health services and, for some, a decline in local care: “Our ER is only open on weekends and is sometimes closed [even then] due to staff shortages or doctor shortages.” Participants from smaller communities noted the vulnerability of a lack of local emergency transport resources:

*Our health care centre doesn’t have community access to ambulance service. Having a heart attack [here] or its environs after hours when the centre is closed is an almost surety that the golden hour will be missed as the ambulances are based in a different community.*

Most of the comments on the urgency of access to emergency health services were made with an awareness of highway traffic and the inevitability of vehicle accidents: “*Being on a main highway with 40,000 vehicles travelling through the mountains per day means numerous vehicle accidents happen which burdens our health care centre and most have to be transported by ambulance 4 hours away*”). Other participants noted an increased vulnerability due to local industry:

*Our emergency is only open 8am-7pm which is awful with 5 mines surrounding us, lots of young families, and a busy highway that closes frequently in the winter, shutting us off from other communities.*

Interview participants echoed many of the same concerns as the survey respondents, relaying in more detail the vulnerability they felt. As one noted, “You don’t feel warm and fuzzy living in a rural area [or] that you are safe and that someone is going to help you if you come into an emergency.” Many respondents cited an awareness that local emergency response times exceeded an acceptable time frame of one hour:

*People have died of a heart attack and haven’t had a response time that would… even be remotely close to what would be needed to support someone with a heart attack.*

Many participants also pointed to the distant locations of ambulance stations exacerbating response times: “By the time an ambulance gets to you it could be 40 minutes or more”. This was at times complicated by inadequate staffing: “Sometimes we don’t even have an ambulance attendant here”.

##### Maternity care

Another thematic concern for survey and interview respondents was a lack of access to local maternity care and the attendant impact on the wider community, namely the inability to attract young families to the area. For those local families who did have to leave their communities to access care, the difficulties were substantial and included financial costs over many weeks (travel, accommodation, lost wages for partners) and risks to mothers and their babies who are required to travel to and from care.

##### Specialist care

Due to low population density and geographic isolation, few rural communities have a full complement of local specialists. Respondents recognized the effects of a lack of local specialist care, including increased difficulty retaining primary care providers and increased need to travel to access specialist services in regional referral communities. Many respondents commented on the challenge of travel to referral sites. Several participants noted that they did not know anyone in the regional centres, making finding accommodation and securing support more difficult. Participants stressed the dire impact of the cancellation of a private bus service that historically linked rural BC communities to one another and to larger centres (i.e., Greyhound). Several participants suggested to have specialists visit rural communities on a regular basis instead of having individual patients travel out of their communities. The concerns regarding a lack of access to specialist care for rural areas were reflected in the interview data as well.

#### Mental health, substance use and addiction services

The importance of access to mental health, substance use and addiction services was cited by many respondents, along with a need for increased local resources. These individuals felt that local services did not reflect population needs, marked by an over-reliance on volunteers and inadequately trained health professionals. As one participant noted,

*I went to see someone for postpartum depression once, and it was just a nurse who basically handed me a booklet on depression. I needed a counselor: we don’t have one.*

According to several participants, the effects of this lack of resources included a rising prevalence of accidental drug overdose.

### The consequences of a lack of access to care

The most significant consequence of a lack of access to local care was treatment avoidance (or missed treatments) due to insurmountable logistics of travel combined with long wait-times. As one respondent noted, *“A lot of people are foregoing necessary medical tests/ scans/ procedures simply because they are unable to afford to leave town”.* The consequences of a lack of access to local care were more acutely felt by those without the financial means to travel nor the social capital for support to reach care.

Interview respondents identified in more detail the challenges of travel to access care outside of their communities for both planned appointments and emergency care. The former focused on the time commitment for often short visits, geographic barriers, and financial and social costs, and the latter focused on how to get back home after receiving care. One participant recalled spending $1,000 to fly to an 11-minute specialist appointment. Another respondent described a time when a friend of theirs was transported out of the community to receive emergency care:

*“His biggest worry must’ve been all the equipment strapped to him, but he was really fretting about how he was going to get home.”*

According to participants, the most difficult situation to navigate was the need for out-of-community chronic or extended care involving repeat visits.

### Suggestions for improvements

Respondents, when prompted, had many suggestions for improving their local healthcare including increasing local capacity to deliver services and mitigating the effects of travel. Each theme is described in more detail, below.

#### Improved local access to healthcare

Most respondents felt that expanding the care available in all health categories noted above was paramount. Stabilizing local care was seen as *preventative*, reducing the need for out-of-community travel. This was seen as important for both local residents, but also primary and allied care providers. As one respondent wrote:

*I think that ultimately if we could treat people in town for more care we wouldn’t be driving half of our ambulance staff down the highway to other communities and splitting our resources.*

Many respondents recognized the need to focus on capacity and facilities development in the present to stabilize care into the future. Suggestions were contextually specific and included increasing the number of beds in a local facility and supplying “more, modern hospital equipment for visiting specialists,” which would potentially lead to increased specialist outreach clinics in smaller communities. Many noted that population growth needed to be addressed in health services planning.

Beyond infrastructure and planning, most respondents felt that stabilizing and growing local primary care was an essential building block to improving healthcare. In some instances, this included re-opening local hospitals and increasing the number of local care providers. Several respondents noted the importance of retention with a focus on new recruits. As one respondent wrote, “Come up with a plan to attract and keep doctors. Stop rotating doctors through our clinics.” Workforce stability was seen as increasingly important in enabling access to emergency care and local maternity care. Many respondents recognized the need for a solution beyond acute care services, however, and suggested walk-in clinics to both increase the efficiency of the local hospital and reduce the burden on family physicians. Several respondents suggested the need for stable care provider contracts and supported local housing. In addition, suggestions were made for system-level incentives (e.g., additional benefits for northern practice, increased remuneration for ambulance attendants).

Many participants noted the importance of improving the capacity of the larger inter-professional healthcare team, beyond physicians. Respondents noted the importance of this to increase efficiency and safety, and to address the chronic shortage of physician providers (e.g., through increasing the scope of First Responders, the integration of Nurse Practitioners, etc.).

Several participants wrote about the need for training for care providers to better interact with elderly patients and for increased accountability in communications within the hospital and between the ER and family physicians. Others noted the importance of removing barriers for International Medical Graduate practice and respondents from the east of the province emphasized the need for a reciprocity agreement with their neighboring province (Alberta).

Almost all participants recognized and prioritized the importance of a community-driven approach to sustainable rural healthcare planning. Practical solutions included re-instituting local hospital boards^3^, creating intentional partnerships between local community organizations and healthcare providers, and moving towards local ownership of facilities. Activities that community members could take on included marketing and advertising the attributes of rural communities to potential care providers. All solutions were understood to be enabled by increased healthcare funding to rural communities. Priority areas noted were infrastructure and health human resources.

 Interview participants expanded on ideas of community participation, most expressing frustration with decision makers’ lack of engagement. Another participant expressed a lack of genuine engagement from their regional Health Authority after participating in a day-long workshop:

*[It] was a waste of time because they had already made up their mind on how they were going to deal with things and I think we were just lip service to say that they did talk to the community.*

Experiences were summed up with the assessment that “We don’t have a voice here”.

#### Mitigating the effects of travel

Many respondents had suggestions for mitigating the effects of travel to access healthcare, including increasing the use of technology to enable care at a distance and support for patients to travel back home after receiving emergency care.

Beyond system-level changes, several respondents noted more simple solutions including mobilizing volunteers to ensure safe return and implementing a system of referral site calling to ensure arrival. There was agreement that travel to access health services – and return home – should be a social imperative for those unable to afford it.

Ultimately, most participants felt that they modulated their expectations of local care to match what was possible and reasonable. Several noted “*we just want the basics*” and *“we don’t expect a small hospital like ours to have CAT scans and PET scans, and all this expensive equipment. But what we do expect is to have reasonable, accessible healthcare for our citizens.”*

## Discussion

Incorporating citizen-patient priorities into health planning processes is gaining traction in policy and research domains [2, 18, 19]. However, concerns remain, including those of a misalignment between citizen-patient and system priorities and the impractical or cost-prohibitive desires of citizens-patients. This means that although citizen-patient priorities may be well-articulated through local and regional outreach mechanisms, they are likely to remain stagnant without a framework for integrated health planning. The two essential prongs of this framework include *merging community-articulated priorities with needs-based planning data* and *system-embedded accountability* for the inclusion of citizen-patient priorities. That is, there needs to be a transparent way of reconciling differences between what a community says that they want and what healthcare planners say that they need. This must be accompanied by an accountability framework; that is, the checks and balances that allow us to measure how well this has been achieved. When taken together, this approach can determine areas for strategic investment.

Needs-based planning has been proposed as a response to supply-side analysis of system utilization [20, 21]. This retrospective planning approach does not allow for quickly changing circumstances nor, most importantly, for the possibility that current service delivery levels may correspond to ‘what is’ and not ‘what is needed’. This not only has the potential to stifle innovation and deter experimentation with new models of care, but it also focuses healthcare planning on *system* needs as opposed to *patient* needs. An alternative model involves identifying the population served and using rational planning methods to anticipate needs. For example, in their needs-based approach for determining substance use services and supports in Canada, the Centre for Addiction and Mental Health described a three-staged process including (1) determining the population catchment (geographic area and size of the population served), (2) establishing need categories and estimating the number of people within each need category, and (3) estimating service needs within each need category [22]. This approach has been implemented in British Columbia in the context of rural maternity service planning. In their paper, Grzybowski et al. [23] described the creation of population catchments surrounding each rural facility in the province that offered maternity care and a process for linking health service outcomes to the geographic catchments through postal codes. This framing allows us to understand not only current utilization, but also to calculate need for services based on volume, isolation and population vulnerability [23]. Integrating citizen-patient priorities into a needs-based planning framework allows health system users to collaborate with administrators. Likewise, it allows for a layering of other considerations including regional and provincial priorities and issues of feasibility. This results in areas for strategic investment.

A persistent challenge for citizen-patient engagement in healthcare planning involves following through after engagement has occurred: the ‘what next?’ [24–26]. Acting on citizen-patient priorities is difficult in a system with a lack of an entrenched mechanism for rigorously integrating citizen-patient voices. This can be addressed through an *accountability framework* that ensures community-level data is taken into consideration. System accountability can be framed through different approaches. In their article, “Thinking about Accountability”, Raisa Deber [27] described *financial accountability* which, in the instance of citizen-patient healthcare priorities, ties program funding to the integration of citizen-patient voices in healthcare decision-making. The author also highlighted the utility of *information* directed towards key stakeholders – in this case, the public – which makes transparent how citizen-patient voices have been integrated into planning through performance measures and improvement [27].

An external and transparent framework is necessary to reflect and validate the extent to which citizen-patient voices are included in health planning. Such an accountability framework would ensure that engagement processes are rigorous and demographic representation (including vulnerable populations) is appropriate. It also requires a move away from reactive engagement on a pre-set topic to ground-up articulation of citizen-patient-identified priorities. This significant shift has already begun, largely driven by demand from citizens-patients themselves supported by health system and research commitments to engage citizens-patients.

Solutions to the health service challenges reported by participants in this study, namely lack of access to local healthcare and difficulties traveling to access care outside of one’s home community, led to thoughtful solutions. These solutions, including expanding the availability of existing local care, increasing the size and capacity of the workforce and facilitating local access to specialist care through outreach clinics and virtual care platforms, are sensible and congruent with the broad planning priorities of the jurisdiction in which the data were collected [4, 28, 29]. In this instance, the study findings provide useful triangulation for healthcare planning decisions and enable a voice to rural citizens.

Finally, it is important to acknowledge that although the survey and interview participants reported what is needed to improve healthcare in their rural communities (deficits-focused) as opposed to what is working well (strengths-focused), this was influenced by the intent and wording of the research questions. This study was conceptualized to provide evidence for areas of rural healthcare improvement. We are confident that, if the research questions had been framed through a lens of appreciative inquiry (positive, strengths-based), narratives of innovation and resilience in rural communities would have come through clearly.

### Limitations

A limitation of the present study includes the choice of online platform for survey delivery. Not all rural regions in British Columbia have access to reliable, high-speed internet services and lack of access is exacerbated for those who are more isolated and financially insecure. Although we attempted to remediate this barrier by offering telephone interviews in place of the online survey, it is unclear to what extent the choice of online platform might have affected participation. However, we also recognized the advantages that a virtual platform allows in terms of breadth of reach, especially where resources are limited, and feel that in this case, the benefits outweighed any limitations.

Additionally, we adopted a voluntary sampling approach to data collection, which means that respondents self-selected into the survey and interviews. The choice of sampling method was pragmatic, allowing for expansive reach across rural British Columbia within a short period of time and at low cost. There are limitations to this approach, however, including that we cannot know how many people decided not to participate (response rate) and whether those who did not respond differed systematically from those who did (non-response bias).

Our sample comprised more female than male respondents (911 and 233, respectively); this is compared to the population of BC, where the proportion of females to males is roughly equal [30]. It is recognized that females are more likely to participate in survey research than males [31, 32]. Nonetheless, the underrepresentation of males in the survey represents a gender bias that might have impacted the findings of the research. Likewise, the mean age of the total sample was 55 years, which is higher than the mean age of the population of BC (42.2 years) [30]. It is possible, then, that the findings of the research are biased to the experiences and priorities of older adults.

Lastly, we did not collect race-based and Indigenous identity data, which might have revealed unique health care priorities across groups and is therefore, another limitation of the study. First Nations, Métis and Inuit in Canada have inherent and collective rights to self-determination, which include ownership and governance of their data. Therefore, there are unique considerations for the collection and use of Indigenous identity data, including for example, community engagement and data governance agreements [33]. To do this appropriately requires resourcing (funding, time) that was beyond the capacity of the project. We acknowledge, though, that a large proportion of Indigenous peoples in BC reside in rural areas (30.3 %) and that their voices are essential to understanding the full extent of rural citizen-patient and community priorities for healthcare in British Columbia [34]. The voices of BC’s rural and remote Indigenous peoples are not appropriately captured in this research.

## Conclusions

The findings of this study demonstrate a clear articulation of healthcare priorities across rural BC, including a need for better access to services for many communities. A secondary output of this research was the proof of concept of the utility of online survey-based data collection for widespread geographic reach at low cost. Soliciting and documenting the healthcare needs and priorities of rural citizens-patients can be achieved through diverse approaches. Survey research in particular is just a ‘snap-shot in time’; in response to often quickly changing circumstances and priorities, ongoing and entrenched avenues for citizen-patient participation in health planning are essential.

## Supplementary Information

**Additional file 1. **Survey Instrument

## Data Availability

The datasets analyzed during the current study are not publicly available to maintain and protect the privacy of study participants, but are available from the corresponding author on reasonable request.

## Abbreviations

BC: British Columbia
BREB: Behavioural Research Ethics Boards
CPC: citizen-patient-community
NZ: New Zealand
RCCbc: Rural Coordination Centre of British Columbia
RSA: Rural Subsidiary Agreement
SPOR: Strategy for Patient-Oriented Research
UBC: University of British Columbia

## Footnotes

1 The British Columbia Patients as Partners initiative aims to ensure that patient, family and caregiver voices are incorporated at all levels of the healthcare system, by providing leadership, training and other resources to support engagement and the integration of patients’ and families’ voices, choices and representation into healthcare system decision-making.

2 The British Columbia RSA, between the Government of BC, Doctors of BC and the Medical Services Commission, is intended to improve the availability and sustainability of physician services in rural and remote areas of BC through targeted funding for provider recruitment, retention and education. Community eligibility for RSA support is measured according to their level of isolation (e.g., community size, distance to a major medical centre, number of general practitioners within 35 kilometres). More information on the RSA, including the full agreement and a list of eligible communities, is available here: https://www2.gov.bc.ca/gov/content/health/practitioner-professional-resources/physician-compensation/rural-practice-programs/rural-practice-subsidiary-agreement.

3 The regionalization of healthcare in British Columbia, beginning in the early 1990s, saw the disbandment of local hospital boards in favour of regional governance structures (“Regional Health Authorities”), which assumed responsibility for the administration and delivery of healthcare services in discrete territorial jurisdictions within the province.
