# Supplementary material for: Rural citizen-patient priorities for healthcare in British Columbia, Canada: findings from a mixed methods study
Source: BMC Health Serv Res. 2021 Sep 18;21:987. doi: 10.1186/s12913-021-06933-z (PMC8449919; doi:10.1186/s12913-021-06933-z)
Supplement: Supplementary file 1 — Additional file 1. Survey Instrument [file 12913_2021_6933_MOESM1_ESM.docx]

Appendix A. Survey Instrument

Q1. Where do you live?

*It helps us to know where you live so that we can understand common issues across communities.*

[open-text response]

Q2. How long have you lived there?

[open-text response]

Q3. What year were you born?

[open-text response]

Q4. I am:

- Female (1)
- Male (2)
- Other (3)
- Prefer not to say (4)

Q5. Are you a paid health care provider?

- Yes (1)
- No (2)

*Display Q6 if ‘Are you a paid healthcare provider?’ = Yes*

Q6. What type of provider are you?

- Registered Nurse (1)
- Nurse Practitioner (2)
- Public Health Nurse (3)
- Other Nurse (4): [please specify]
- Midwife (5)
- Allied Health Professional (e.g., Massage Therapist, Chiropractor) (6)
- Family Physician (7)
- Other Physician (8): [please specify]

Q7. In your opinion, what are the most important health care priorities in your community? Please be specific.

[open-text response]

Q8. Why are these priorities important in your community?

[open-text response]

Q9. What do you think could be done to improve health care in your community?

[open-text response]

Q10. Is there anything else that you would like to share with us?

[open-text response]
